# Curcumin protects retinal pigment epithelial cells against oxidative stress via induction of heme oxygenase-1 expression and reduction of reactive oxygen

**Published:** 2012-04-11

**Authors:** Je Moon Woo, Da-Yong Shin, Sung Ju Lee, Yeonsoo Joe, Min Zheng, Jin Ho Yim, Zak Callaway, Hun Taeg Chung

**Affiliations:** 1 Department of Ophthalmology, Ulsan University Hospital, Ulsan, Republic of Korea; 2 School of Biological Sciences, University of Ulsan, Ulsan, Republic of Korea; 3 University of Ulsan College of Medicine, Ulsan, Republic of Korea

## Abstract

**Purpose:**

To determine whether curcumin induces expression of the defensive enzyme heme oxygenase-1 (HO-1) and protects cells against oxidative stress in cultured human retinal pigment epithelial cells.

**Methods:**

Effective concentrations and toxicities of curcumin were determined after 3 h of curcumin treatment with the 3-(4,5-dimethylthiazol-2-yl)-2,5-diphenyltetrazolium bromide assay. Confluent human retinal pigment epithelium cell lines (ARPE-19) were preincubated with curcumin and oxidatively challenged with H_2_O_2_. HO-1 expression was determined with western blot analysis. To confirm the protective role of HO-1 in oxidative stress, small interfering RNA (siRNA) against HO-1 or inhibitor of HO-1 was treated with curcumin in retinal pigment epithelium cells. Intracellular levels of reactive oxygen species (ROS) were measured with flow cytometry and confocal microscopy. Apoptosis was evaluated with Annexin V-fluoroscein isothiocyanate staining.

**Results:**

Curcumin had little cytotoxicity at concentrations less than 30 μM, and HO-1 expression was the highest at the 15 μM concentration. At this concentration, curcumin also increased the cytoprotective effect against the oxidative stress of H_2_O_2_ through the reduction of ROS levels in human retinal pigment epithelial cells. Curcumin’s effect on the reduction of ROS was mediated by the increase in HO-1 expression.

**Conclusions:**

Curcumin upregulated the oxidative stress defense enzyme HO-1 and may protect human retinal pigment epithelial cells against oxidative stress by reducing ROS levels.

## Introduction

Age-related macular degeneration (AMD) is the most common cause of blindness in patients aged 65 or over in the Western world [1], and incidence continues to rise as a result of the increasing percentage of older adults in the general population. Pathologically, AMD results from retinal pigment epithelium (RPE) dysfunction or loss associated with photoreceptor fallout, Bruch’s membrane thickening, and choriocapillary hypoperfusion [2]. The RPE is a monolayer of pigmented cells forming part of the blood retina barrier and is particularly susceptible to oxidative stress because of the layer’s high consumption of oxygen. Thus, chronic oxidative stress induces RPE damage that is responsible for the aging process and may therefore play an important role in the pathogenesis of AMD [3,4]. Human RPE has many antioxidative enzymes such as superoxide dismutase, heme oxygenase, and enzymes involved in glutathione synthesis [5,6]. Heme oxygenase-1 (HO-1) is a ubiquitous and redox-sensitive inducible stress protein known to protect cells against various types of stress. The importance of this protein in physiologic and pathological states is underlined by the versatility of HO-1 inducers and the protective effects attributed to heme oxygenase byproducts in conditions associated with moderate or severe cellular stress [7,8].

Curcumin, a biologically active component of turmeric, which has been used in India for medical purposes for centuries, has a variety of pharmacological activities, including antioxidant, anti-inflammatory, and antiproliferative effects. Curcumin is an effective scavenger of reactive oxygen species in vitro and indirectly enhances the synthesis of antioxidative enzymes [9,10]. In this study, we hypothesized that curcumin has cytoprotective effects with HO-1 expression against H_2_O_2_ oxidative stress in cultured human retinal pigment epithelial cells.

## Methods

### Materials

Curcumin, H_2_O_2_, zinc protoporphyrin (ZnPP; HO-1 inhibitor), cobalt protoporphyrin (CoPP; HO-1 stimulator), and SB 203580 were purchased from Sigma Aldrich (St. Louis, MO). 3-(4,5-dimethylthiazol-2-yl)-2,5-diphenyltetrazolium bromide (MTT) and 2’7’-dichlorodihydro-fluorescein diacetate (H2DCFDA) were obtained from Invitrogen Molecular Probes, Inc. (Carlsbad, CA).

### Cell culture

ARPE-19 cells originated from human retinal pigment epithelial cells. The ARPE cells were purchased from the American Type Culture Collection (ATCC, Manassas, VA). RPE cells were cultured in T-75 flasks with Dulbecco’s modified Eagle’s medium (Invitrogen) supplemented with 10% fetal bovine serum (FBS; Sigma, St. Louis, MO) and 100 U⁄ml penicillin and streptomycin (Gibco-BRL, Gaithersburg, MD). During incubation, the culture medium was changed every 2 days. All cultures were maintained at 37 °C under 5% CO_2_ with 95% relative humidity.

### Cell viability assay (3-(4,5-dimethylthiazol-2-yl)-2,5-diphenyltetrazolium bromide assay)

The MTT assay was used to determine cell viability. Briefly, cells grown in 96-well plates were washed twice with Phosphate buffer solution (PBS; 1.54 mM KH_2_PO_4_,155.17 mM NaCl, 2.71 mM Na_2_HPO_4_-7H_2_O) and replaced with culture medium containing 0.5 μl/ml MTT. After 4 h incubation with MTT solution, medium was carefully removed from the plate, and isopropanol was added to solubilize formazan produced from MTT by viable RPE cells. The absorbance at 540 nm was measured using a microplate reader (Spectromax 190; Molecular Devices Corp., Sunnyvale, CA).

### Western blot analysis

Western blot analysis was used to evaluate HO-1 expression. Cells grown in 6-well plates were washed with PBS twice and replaced with 1 ml of PBS. After RPE cells were harvested on the floor of the plate, cells were lysed using RIPA buffer (Sigma) supplemented with a protease inhibitor cocktail (Roche, Indianapolis, IN). Cell lysates were centrifuged at 13,000× g for 15 min, and bicinchoninic (BCA) protein assay (Pierce, Rockford, IL) was used to determine the protein concentrations of the extracted solutions. Protein concentration was determined with BCA assay. The sample (10 μg protein) was loaded in 10% sodium dodecyl sulfate PAGE gel. Gel proteins were electrophoretically transferred to a poly (vinylidene difluoride) membrane (Thermo Scientific, Rockford, IL). Membranes were blocked with 3% non-fat dried milk in PBS, and then probed with a polyclonal rabbit anti-HO-1 antibody (1:100 dilution in Tris-buffered saline, pH 7.4) overnight in a dark cold room. After three washings with PBS containing 0.05% (v/v) Tween-20, the blots were visualized using an amplified alkaline phosphatase kit from Sigma.

### Transfection of single interfering RNAs

RPE cells were transfected with double-stranded single interfering RNAs (siRNAs; 50 nmol/ml) against *HO-1* (Qiagen, Valencia, CA: target sequence, 5′-CAG GCA ATG GCC TAA ACT TCA-3′ Hs_HMOX1_5) for 12 h with the Lipofectamine method according to the manufacturer’s protocol (Invitrogen, Carlsbad, CA) and recovered in fresh media containing 10% FBS for 12 h. In brief, after combination 2 µg DNA in 100 µl OPTI-MEM and 6 µl Lipofectamine in 100 µl OPTI-MEM, the mixture was incubated at room temperature for 30min and then overlaid the cells. HO-1 expression interference was confirmed with immunoblot using anti-HO-1 antibodies; scrambled siRNA was used as a control.

### Determination of intracellular reactive oxygen species levels

ROS levels were measured using 2’7’-dichlorodihydro-fluorescein diacetate (carboxy-H2DCFDA), which is converted into a non-fluorescent derivative (carboxy-H2DCF) by intracellular esterases. Carboxy-H2DCF is membrane impermeant oxidized to fluorescent derivative carboxy-DCF by intracellular ROS. After the indicated treatments, cells were washed with PBS and incubated with PBS containing 10 μM carboxy-H2DCFDA for 20 min at 37 °C. Loading buffer was removed, and a short recovery time was allowed for the cellular esterases to hydrolyze the acetoxymethyl ester or acetate groups and render the dye responsive to oxidation. Then the cells were returned to prewarmed growth medium and incubated for 20 min at 37 °C. Cells were washed twice with PBS, trypsinized, and collected in 1 ml PBS. Cells were then transferred to polystyrene tubes with cell-strainer caps (Falcon, Becton Dickinson, Franklin Lakes, NJ) and analyzed with flow cytometry. Geometric distributions of carboxy-DCF fluorescence were calculated using FACS (FACScan; Becton Dickinson) and Cell Quest 3.2 (Becton Dickinson) software for acquisition and analysis. Green ROS fluorescence was visualized using an Olympus (Tokyo, Japan)Flouview-FV300 Laser Scanning Confocal system.

### Annexin V-fluoroscein isothiocyanate staining to evaluate apoptosis

Following experimental treatments, cells were centrifuged at 1,000× g for 5 min and the pellets were washed twice with 1.0 ml cold PBS and 1.0 ml cold binding buffer (10 mM HEPES/NaOH, pH 7.4, 140 mM NaCl, and 2.5 mM CaCl_2_). Cells were resuspended in 100 μl of binding buffer and stained with 2 μl of Annexin V-fluoroscein isothiocyanate (V-FITC) solution and 10 μl propidium iodide (PI) solution for 15 min at room temperature in the dark. Samples were then diluted with 400 μl binding buffer and analyzed with flow cytometry within 30 min. A minimum of 50,000 cells were maintained for all samples. Samples were analyzed with a FACScalibur flow cytometer (BD Biosciences, Erembodegem, Belgium).

## Results

### Effect of curcumin on cytotoxicity in human retinal pigment epithelial cells

To evaluate cell viability in different concentrations of curcumin and determine its non-cytotoxic concentration, curcumin dissolved in dimethyl sulfoxide (DMSO) was mixed with variable concentrations of culture medium. RPE cells were plated onto 96-well plates at a density of 2×10^4^/well, and each well contained 200 μl culture medium. After 24 h incubation, RPE cells were incubated with different concentrations of curcumin-containing cell medium for 3 h.

**Figure 1:**
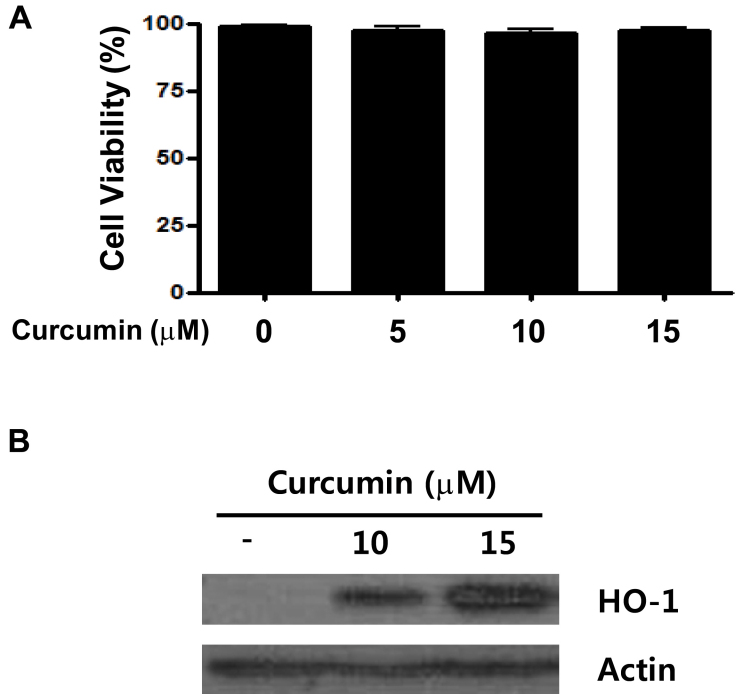
Cell viability and heme oxygenase-1 (HO-1) expression in human retinal pigment epithelial cells (ARPE-19) with different concentrations of curcumin-containing medium. ARPE-19 cells were exposed to various concentrations of curcumin for 3 h. Cell viability (**A**) was measured with 3-(4,5-dimethylthiazol-2-yl)-2,5-diphenyltetrazolium bromide (MTT) assay and HO-1 protein expression (**B**) was measured with western blot analysis. The non-treated and curcumin-treated groups showed no significant difference in cell viability, and HO-1 expression was highest in 15 μM curcumin.

Cell viability was measured with the MTT assay. The non-treated and curcumin-treated groups showed no significant difference in cell viability (Figure 1A).

### Effect of curcumin on heme oxygenase-1 expression in human retinal pigment epithelial cells

To evaluate HO-1 expression of RPE cells under curcumin treatment, RPE cells were plated onto 96-well plates at a density of 3×10^5^/well. After 24 h incubation, cell medium was exchanged with curcumin-containing cell medium and incubated for 3 h. HO-1 expression was evaluated with western blot analysis. HO-1 expression in RPE cells was highest at the 15 μM curcumin concentration (Figure 1B).

### Cell viability of human retinal pigment epithelial cells under oxidative stress by H_2_O_2_

**Figure 2:**
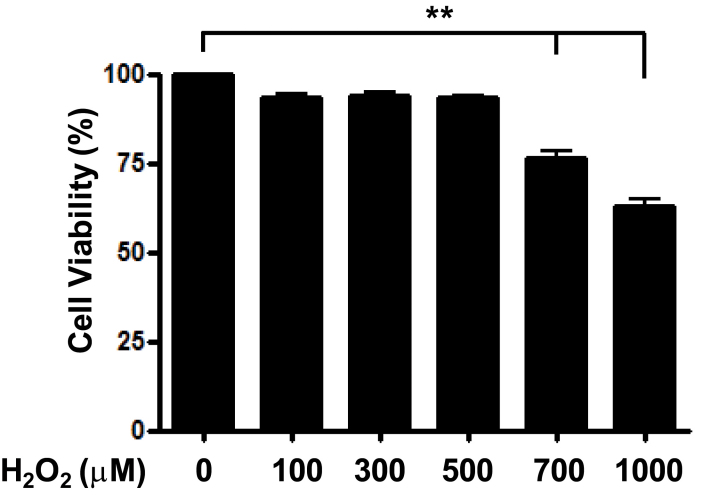
Cell viability in human retinal pigment epithelial cells with different concentrations of H_2_O_2_-containing medium. Human retinal pigment epithelial cells (ARPE-19) cells were exposed to various concentrations of H_2_O_2_ for 3 h. Cell viability was measured with 3-(4,5-dimethylthiazol-2-yl)-2,5-diphenyltetrazolium bromide (MTT) assay. No significant difference in cell viability was observed in doses of less than 500 μM H_2_O_2_. However, doses above 500 μM H_2_O_2_ caused dose-dependent retinal pigment epithelium (RPE) cell death. **p<0.01, ***p<0.001. Histograms are representative of three independent experiments. Each bar represents mean±SD (standard deviation) from three independent experiments.

To evaluate cell viability in different H_2_O_2_ concentrations, H_2_O_2_ was mixed with culture medium. RPE cells were plated onto 96-well plates at a density of 2×10^4^/well, and each well contained 200 μl of culture medium. After 24 h incubation, cell medium was exchanged with H_2_O_2_-containing cell medium of different concentrations, and cells were incubated for 24 h. Cell viability was measured with MTT assay. Concentrations of less than 500 μM H_2_O_2_ showed no significant difference in cell viability; however, in concentrations of more than 500 μM H_2_O_2_, dose-dependent RPE cell death was observed (Figure 2).

### Protective effect of curcumin on retinal pigment epithelial cells against H_2_O_2_-induced oxidative stress via heme oxygenase-1 expression

**Figure 3:**
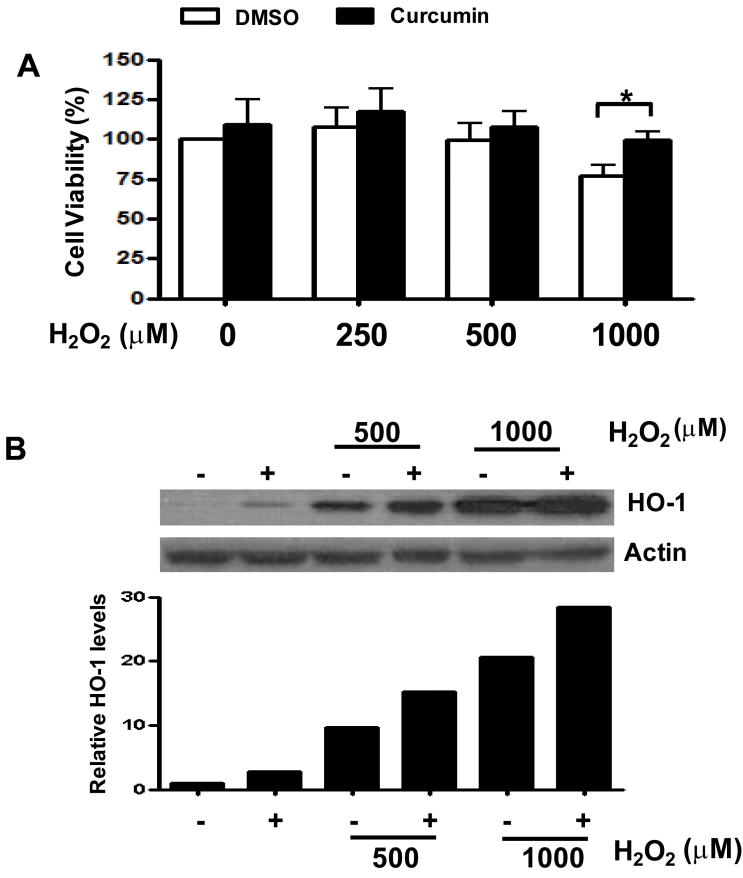
Cell viability and heme oxygenase-1 (HO-1) expression in human retinal pigment epithelial cells between curcumin pretreatment and control dimethyl sulfoxide (DMSO) groups with various H_2_O_2_ concentrations. **A**: Cell viability measured with 3-(4,5-dimethylthiazol-2-yl)-2,5-diphenyltetrazolium bromide (MTT) assay. Human retinal pigment epithelial cells (ARPE-19) cells pretreated with 15 μM curcumin showed more cell viability than those from the control DMSO group. The difference was highest in 1000 μM H_2_O_2_. *p<0.05 . The percentage of live cells among the total cells was calculated from three separate experiments. **B**: HO-1 protein expression was measured with western blot analysis. The plus sign means pretreatment with 15 μM curcumin. HO-1 expression was higher in the curcumin-pretreated group under treatment of H_2_O_2_ compared to the control group. β-actin served as the standard. Results were quantified with densitometry. The image for HO-1 was shown from more than three separate experiments.

RPE cells in 96-well plates were incubated for 24 h and pretreated with 15 μM curcumin for 3 h. Each well was then washed with PBS, and different concentrations of H_2_O_2_ (0, 500, and 1,000 μM) were added for an additional 3 h. Cell viability was measured with the MTT assay, and HO-1 protein expression was measured with western blot analysis. Curcumin pretreatment showed a cytoprotective effect against H_2_O_2_ oxidative stress in RPE cells. Cell viability in the curcumin-pretreatment group was higher than in the control DMSO group. At higher H_2_O_2_ concentrations, cell viability decreased dose-dependently as in the previous results. At the 1,000 μM H_2_O_2_ concentration, the curcumin-pretreatment and control DMSO groups showed the greatest significant difference in cell viability (Figure 3A). HO-1 expression in the curcumin-pretreatment group was higher than in the control group (Figure 3B).

### Effect of curcumin on reactive oxygen species reduction via stimulating heme oxygenase-1 expression

**Figure 4:**
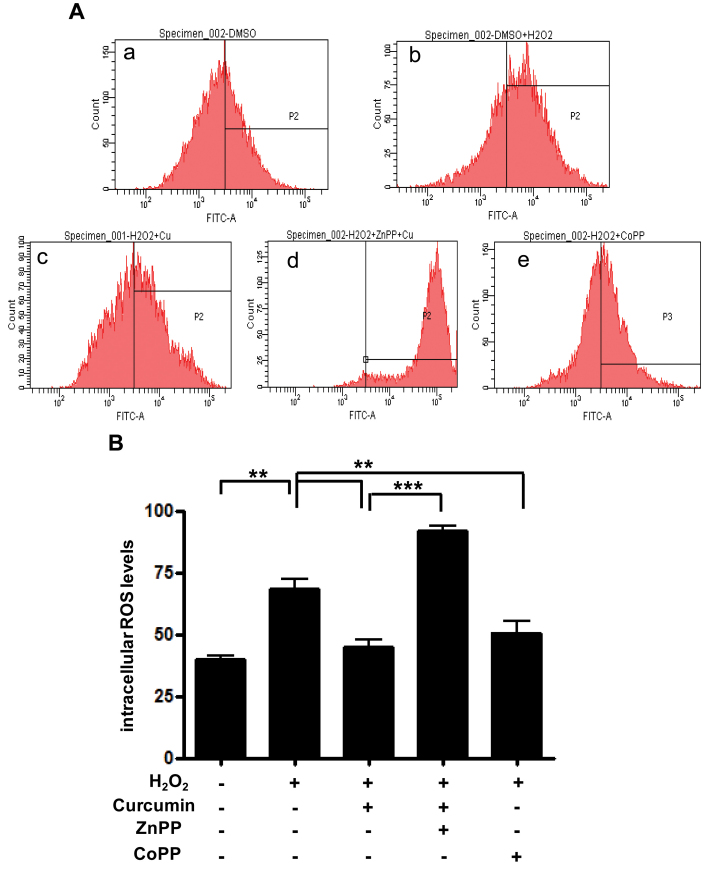
Effect of curcumin on intracellular reactive oxygen species (ROS) levels. Retinal pigment epithelium (RPE) cells were separately pretreated with 15 µM curcumin and 10 µM CoPP known as heme oxygenase-1 (HO-1) stimulator for 3 h. About 10 µM ZnPP, a known HO-1 inhibitor, was added to cells preprocessed for 1 h after 2 h pretreatment with 15 µM curcumin. Cells were then treated with 1000 µM H_2_O_2_ for 3 h. Intracellular ROS levels in RPE cells were measured with flow cytometry (see Materials and Methods). In contrast, ROS levels were increased (shift in relative fluorescence intensity) in cells cotreated with curcumin and ZnPP. **A**: (a) dimethyl sulfoxide (DMSO), (b) DMSO + H_2_O_2_, (c) curcumin (15 µM) + H_2_O_2_, (d) curcumin (15 µM) + H_2_O_2_ +ZnPP (10 µM), (e) CoPP (10 µM) + H_2_O_2_. **B**: Columns, percentage of DCF-CA-stained cells for indicated conditions. Each bar represents the mean±SD of three independent experiments (**p<0.01, ***p<0.001)

**Figure 5:**
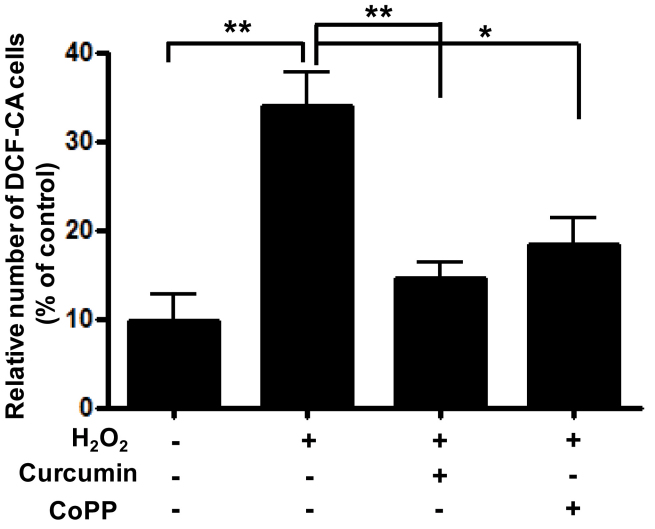
Effect of curcumin on reactive oxygen species (ROS) generation in retinal pigment epithelium (RPE) cells. Cells were separately pretreated with curcumin (15 μM) and CoPP as a heme oxygenase-1 (HO-1) activator for 3 h and then treated with 1000 μM H_2_O_2_ for 3 h. ROS levels treated with curcumin and those treated with CoPP decreased at a similar rate. To quantify cells with increased intracellular ROS levels, the percentages of fluorescent cells among the total cells were calculated and the means± SD were expressed from three separate experiments. *p<0.05, **p<0.01.

**Figure 6:**
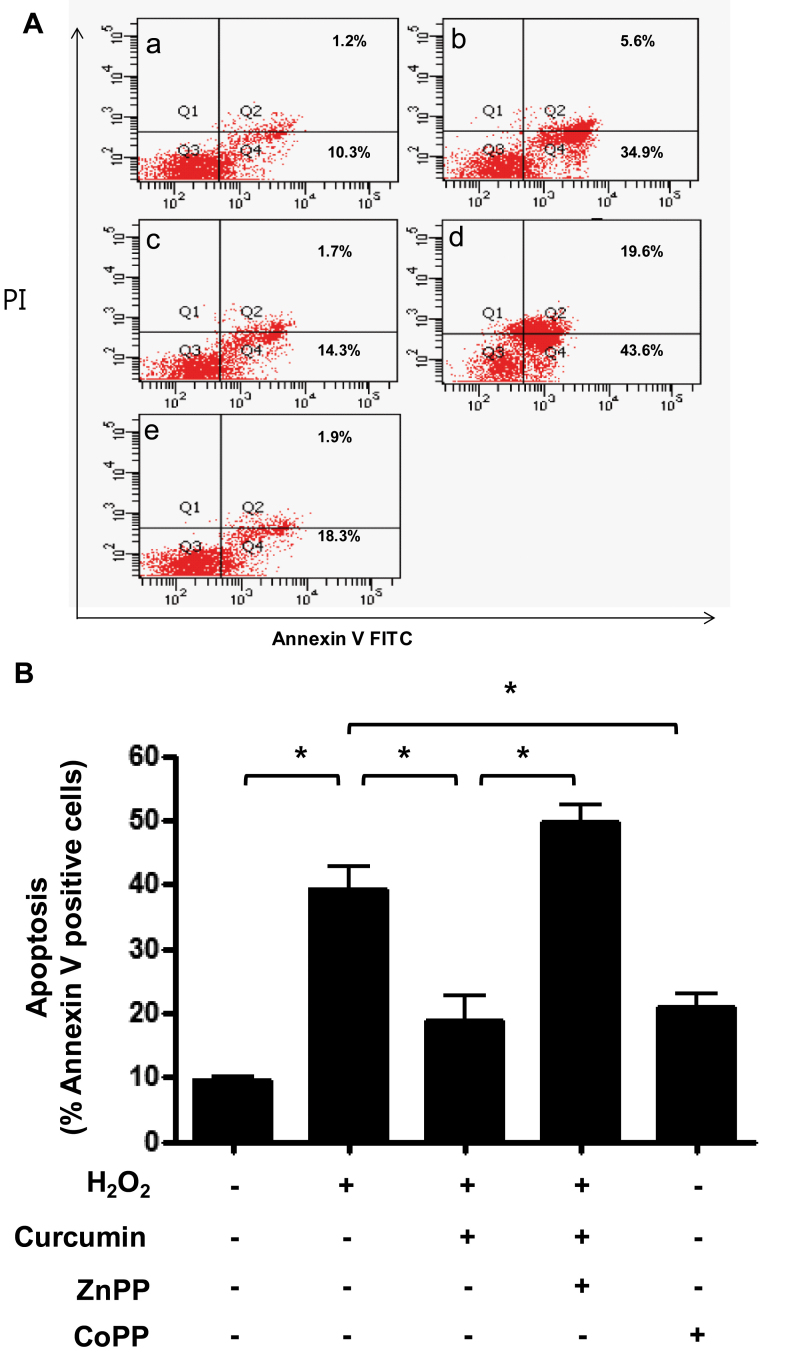
Effect of curcumin on H_2_O_2_-induced apoptosis. Retinal pigment epithelium (RPE) cells were pretreated with 15 µM curcumin and 10 µM CoPP for 3 h. About 10 µM ZnPP was added to cells preprocessed for 1 h after 2 h pretreatment with 15 µM curcumin. Cells were then treated with 1000 µM H_2_O_2_ for 3 h. Apoptotic cells were detected with Annexin V-FITC staining and analyzed with flow cytometry. **A**: (a) dimethyl sulfoxide (DMSO), (b) DMSO + H_2_O_2_, (c) curcumin (15 µM) + H_2_O_2_, (d) curcumin (15 µM) + H_2_O_2_ +ZnPP (10 µM), (e) CoPP (10 µM) + H_2_O_2_. **B**: Columns, percentage of Annexin V positive cells for indicated conditions. Each bar represents the mean±SD of three independent experiments (*p<0.05)

RPE cells were plated onto 12-well plates at a density of 3×10^5^/well, and each well contained 2 ml of culture medium. After 1 day of incubation, some cells were pretreated with either DMSO, 15 μM curcumin, or 10 μM CoPP (as an HO-1 stimulator) for 3 h, and other cells were pretreated with 10 μM ZnPP (as an HO-1 inhibitor) for 1 h and 15 μM curcumin for 3 h. After different pretreatments, all cells were treated with 1,000 μM H_2_O_2_ for 3 h. ROS levels were determined using 10 μM carboxy-H2DCFDA with flow cytometry and confocal microscopy. In the cytometry fluorescence histograms, H_2_O_2_ caused the ROS levels to increase compared to the control group. At the 1,000 μM H_2_O_2_ concentration, 15 μM curcumin reduced ROS levels compared with DMSO. The result for 10 μM CoPP as an HO-1 stimulator was similar to that for 15 μM curcumin. Pretreatment with ZnPP reversed curcumin’s reduction of ROS. These findings suggest that curcumin reduces ROS levels by stimulating oxidative defense enzyme production such as HO-1 (Figure 4A,B). In confocal microscopy, the extent of staining in the 1,000 μM H_2_O_2_–treated group appeared to be greater than in the control group. ROS levels in the 15 μM curcumin–treated group or 10 μM CoPP–treated group appeared lower than in the 1,000 μM H_2_O_2_–treated group. The results from confocal microscopy were similar to the findings from of the cytometry analysis (Figure 5). To evaluate apoptosis under the same conditions as ROS measurement, Annexin V/PI staining and flow cytometry were used. As expected, the effects of curcumin on H_2_O_2_-induced apoptosis corresponded with the ROS measurement (Figure 6A,B). To further confirm the inhibitory effect of curcumin on apoptosis via HO-1, RPE cells were transfected with siRNA.

**Figure 7:**
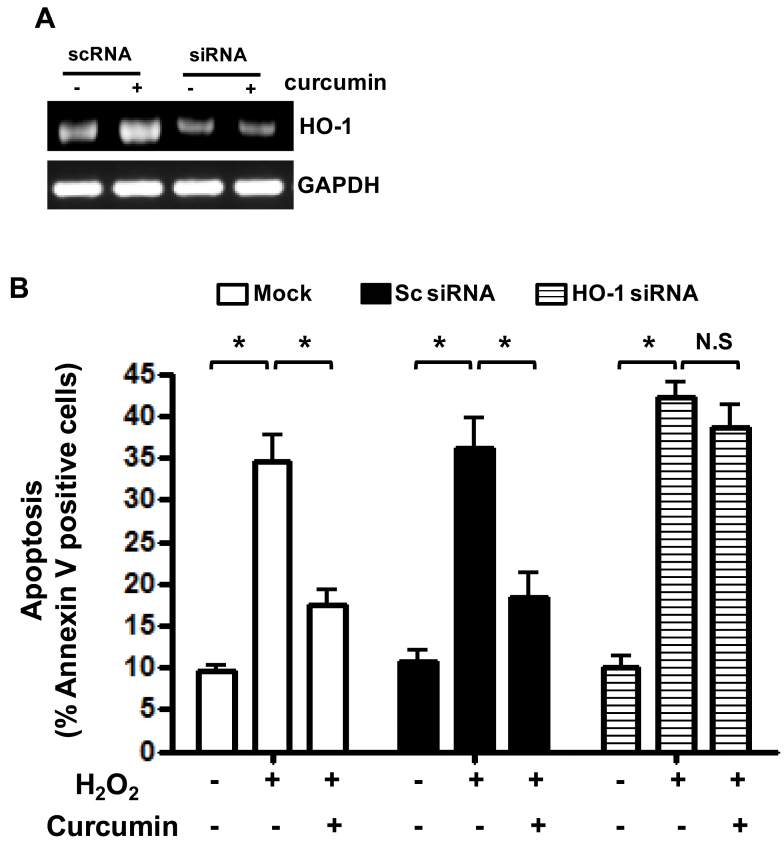
Involvement of heme oxygenase-1 (HO-1) in H_2_O_2_-induced apoptosis in retinal pigment epithelium (RPE) cells. Human RPE cells were transfected with control single interfering RNA (siRNA) or *HO-1* siRNA. After being pretreated with 15 μM curcumin for 3 h, cells were treated with 1000 µM of H_2_O_2_ for 3 h. **A**: Transfection with siRNA against HO-1 caused reduction in HO-1 expression. The expression levels of HO-1 were determined by Western immunoblot analysis **B**: Apoptotic cells were detected with Annexin V-FITC staining and analyzed with flow cytometry. Histograms are representative of three independent experiments. Each bar represents mean±SD (standard deviation) from three independent experiments. *p<0.05.

**Figure 8:**
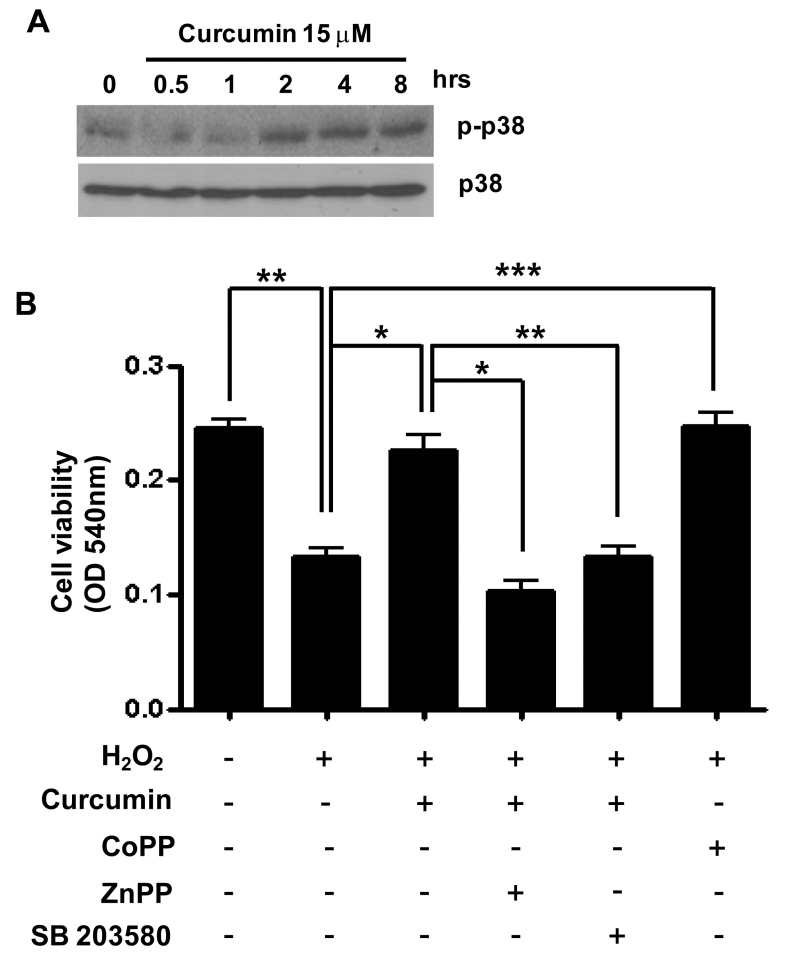
Role of p38 activation in induction of heme oxygenase-1 (HO-1) by curcumin. **A**: Retinal pigment epithelium (RPE) cells were treated with 15 μM curcumin at the indicated time point. Phosphorylated p38 and p38 levels were detected with western blotting. **B**: RPE cells were pretreated with 15 µM curcumin and 10 µM CoPP for 3 h. About 10 µM ZnPP or 20 µM SB203580 as a p38 inhibitor was added to cells preprocessed for 1 h after 2 h pretreatment with 15 µM curcumin. Cells were then treated with 1000 µM H_2_O_2_ for 3 h. Cell viability was determined with 3-(4,5-dimethylthiazol-2-yl)-2,5-diphenyltetrazolium bromide (MTT) assay. Data are expressed as mean±SEM from four independent experiments. *p<0.05, **p<0.01, ***p<0.001.

As shown in Figure 7, in the absence of HO-1, curcumin could not suppress H_2_O_2_-induced apoptosis. Therefore, we suggest HO-1 induced by curcumin plays an important role in H_2_O_2_-induced apoptosis in RPE cells. In an effort to understand the possible mechanism(s) responsible for HO-1 expression and reduction of apoptosis by curcumin in RPE cells, we investigated whether curcumin could induce p38 activation. Activation of p38 was increased by curcumin in a time-dependent manner (Figure 8A). Inhibition of p38 significantly reduced the protective effect of curcumin against H_2_O_2_-induced death (Figure 8B).

## Discussion

Aging has been defined as the progressive accumulation of changes with time associated with or responsible for ever-increasing susceptibility to disease and death. The free radical theory of aging proposes that aging and age-related disorders are the result of cumulative damage arising from reactions involving ROS. ROS cause oxidative damage to cell cytoplasmic and nuclear elements and cause changes to the extracellular matrix [11,12]. The retina is particularly susceptible to oxidative stress because of its high consumption of oxygen. The role of oxidative stress in the pathogenesis of AMD is biologically important. Much of the research into the relationship between oxidative stress and AMD has supported the hypothesis that there is a link between oxidative events and the onset of AMD [3,4,13]. Human studies show that dietary intake and supplementation with antioxidants, such as glutathione, vitamin C, superoxide dismutase, catalase, vitamin E, carotenoids, and zinc, are associated with reduced risk of AMD [14,15]. Other studies show that flavonoids commonly found in fruit and vegetables provide short- and long-term protection, and long-term protection is associated with phase-2 proteins such as glutathione metabolism and HO-1 [16].

Curcumin, the natural yellow pigment phenolic compound contained in turmeric and isolated from the rhizomes of the plant *Curcuma longa*, is generally regarded as the most active constituent of turmeric [17]. In the Ayurvedic system of medicine, turmeric is used as a tonic and blood purifier. Its role in skin disease treatment and its ability to soften rough skin resulted in prolific use in topical creams and bath soaps in India. Turmeric’s use as an anti-inflammatory and antimicrobial agent has been recognized for more than a century [18,19]. Several animal studies have shown that curcumin used in painting colors and flavoring in food possesses anti-inflammatory and antioxidant properties and affords protection against chemical carcinogen-induced tumorigenesis in skin, colon, stomach, duodenal, lung, and prostate cancer [20,21].

Particularly, curcumin exhibits strong antioxidant activity comparable to vitamins C and E. Curcumin is a potent scavenger of ROS, including superoxide anion radicals, hydroxyl radicals, and ROS, in in vitro studies [9,10]. In addition to direct antioxidant activity, curcumin may function indirectly as an antioxidant by enhancing antioxidant enzymes, such as heme oxygenase, glutathione peroxidase, glutathione reductase, catalase, and phase-2 enzymes such as glutathione S transferase and quinone reductase [22-25]. HO-1 and glutathione S transferase are found in the human retina and can be a defense against oxidative stress. In AMD, the ability to upregulate HO-1 and glutathione S transferase diminishes; as a consequence, oxidative stress increases in human RPE.

HO-1 is the rate-limiting first step in heme catabolism that degrades heme to carbon monoxide (CO), free Fe^2+^, and biliverdin that is further converted to the antioxidant bilirubin by biliverdin reductase [26,27]. Bilirubin also modulates cell signal transduction pathways relevant to inflammation. Free Fe^2+^ rapidly induces ferritin expression and the ATPase Fe^2+^-secreting pump to decrease Fe^2+^, thereby limiting oxidative damage created via the Fenton reaction. CO, the third product of HO-1 activity, stimulates cell signaling similar to nitric oxide, but absent its radical activity [28-32]. CO mediates vasodilatation, inhibits platelet aggregation, and suppresses cytokine production—all factors associated with the amelioration of AMD pathophysiology. Thus, HO-1 induction may confer protection in the retina by increasing resistance to oxidative stress, inflammation, and apoptosis.

Curcumin at higher doses is cytotoxic but at lower doses can exert an adaptive stress response. A pretreatment dose of 20 μM for more than 24 h can induce cell death, but we used doses of 15 μM curcumin for only 3 h, which is below the cytotoxic level. About 15 μM curcumin induced the most HO-1 expression, reduced ROS via HO-1, and exerted significant protection against increasing doses of H_2_O_2_. We propose that p38 activity is important in the signal mechanism for induction of HO-1 by curcumin in RPE cells (Figure 8B). The proposed mechanism is consistent with experiments performed using the garlic extract diallyl sulfide as an HO-1 inducer [33,34].

In vivo models and human clinical trials of high-dose curcumin consumption confirmed the lack of significant toxicity and adverse effects [35-38]. However, absorption, biodistribution, metabolism, and elimination studies of curcumin have unfortunately shown only poor absorption, rapid metabolism, and elimination, which represent the low oral bioavailability of this interesting polyphenolic compound [39-42]. Nevertheless, curcumin is known to pass through the blood-brain barrier, and dietary curcumin was found to be effective against forebrain ischemia and traumatic brain injury in animal models. Furthermore, dietary curcumin has been associated with reduced expression of oxidative stress and inflammation markers in the cerebral cortex in a transgenic mouse model of Alzheimer disease [43].

Recently, dietary supplementation of curcumin has been shown to be effective in modulating redox status in a rat model of streptozotocin-induced diabetic retinopathy and retinal neuroprotection using an in vivo model of light-induced retinal degeneration in rats [44,45].

We do not know how much diet-supplemented curcumin reaches the human retina. However, according to recent use of flavonoids and anthocyanins that express HO-1 for AMD, we believe that curcumin, a strong HO-1 inducer, is a good antioxidant for preventive and augmentative therapy of AMD.
